# The proportion of weight gain due to change in fat mass in infants with vs without rapid growth

**DOI:** 10.1038/s41430-024-01534-5

**Published:** 2024-11-05

**Authors:** William Johnson, Lukhanyo H. Nyati, Shabina Ariff, Tanvir Ahmad, Nuala M. Byrne, Leila I. Cheikh Ismail, Caroline S. Costa, Ellen W. Demerath, Divya J. Priscilla, Andrew P. Hills, Rebecca Kuriyan, Anura V. Kurpad, Cornelia U. Loechl, M. Nishani Lucas, Ina S. Santos, Christine Slater, V. Pujitha Wickramasinghe, Shane A. Norris, Alexia J. Murphy-Alford, On behalf of the Multicenter Infant Body Composition Reference Study (MIBCRS), Lukhanyo H. Nyati, Shabina Ariff, Tanvir Ahmad, Nuala M. Byrne, Leila I. Cheikh Ismail, Caroline S. Costa, Divya J. Priscilla, Andrew P. Hills, Rebecca Kuriyan, Anura V. Kurpad, Cornelia U. Loechl, M. Nishani Lucas, Ina S. Santos, Christine Slater, V. Pujitha Wickramasinghe, Shane A. Norris, Alexia J. Murphy-Alford

**Affiliations:** 1 School of Sport, Exercise and Health Sciences, Loughborough University, Loughborough, UK; 2 SAMRC Developmental Pathways for Health Research Unit, Department of Pediatrics, University of the Witwatersrand, Johannesburg, South Africa; 3 Faculty of Community and Health Sciences, University of Western Cape, Cape Town, South Africa; 4 Department of Pediatric and Child Health, Medical College, The Aga Khan University, Karachi City, Sindh Pakistan; 5 Life Science Group, Isotope Application Division, Pakistan Institute of Nuclear Science and Technology (PINSTECH), Nilore, Islamabad Pakistan; 6 School of Health Sciences, College of Health and Medicine, University of Tasmania, Tasmania, Australia; 7 Department of Clinical Nutrition and Dietetics, College of Health Sciences, University of Sharjah, Sharjah, UAE; 8 Nuffield Department of Women’s & Reproductive Health, University of Oxford, Oxford, UK; 9 Post-graduate Program in Epidemiology, Faculty of Medicine, Federal University of Pelotas, Pelotas, Brazil; 10 Division of Epidemiology and Community Health, University of Minnesota, School of Public Health, Minneapolis, MN USA; 11 St John’s Research Institute, Bengaluru, India; 12 Division of Human Health, Department of Nuclear Sciences and Applications, International Atomic Energy Agency, Vienna, Austria; 13 Department of Paediatrics, Faculty of Medicine, University of Colombo, Colombo, Sri Lanka; 14 School of Human Development and Health, University of Southampton, Southampton, UK

**Keywords:** Ageing, Risk factors

## Abstract

**Background:**

There is extensive evidence that rapid infant weight gain increases the risk of childhood obesity, but this is normally based on childhood body mass index (BMI) only and whether or not this is because infants with rapid weight gain accrue greater fat mass is unknown.

**Objective:**

The primary objective of our study was to test whether the proportion of infant weight gain due to concurrent increases in fat mass is greater in infants with rapid weight gain as compared to those with normal growth.

**Methods:**

Body composition was assessed by (1) air-displacement plethysmography (ADP) at 0 and 6 months in 342 infants from Australia, India, and South Africa and (2) deuterium dilution (DD) at 3 and 24 months in 555 infants from Brazil, Pakistan, South Africa, and Sri Lanka. Weight gain and length growth were each categorized as slow, normal, or rapid using cut-offs of <−0.67 or >+0.67 Z-scores. Regression was used to estimate and contrast the percentages of weight change due to fat mass change.

**Results:**

Approximately 40% of the average weight gain between 0 and 6 months and 20% of the average weight gain between 3 and 24 months was due to increase in fat mass. In both samples, compared to the normal group, the proportion of weight gain due to fat mass was higher on average among infants with rapid weight gain and lower among infants with slow weight gain, with considerable individual variability. Conversely, slow and rapid length growth was not associated with differential gains in fat mass.

**Conclusions:**

Pediatricians should monitor infant growth with the understanding that, while crossing upward through the weight centiles generally is accompanied by greater adiposity gains (not just higher BMI), upward crossing through the length centiles is not.

## Introduction

Many countries recommend using the WHO Growth Standards to identify infants with suboptimal growth [1]. The American Academy of Pediatrics (AAP) recommend using the 2.3rd and 97.7th percentiles [2]. Less guidance, however, is normally provided to identify infants based on change in their percentile position. One of the AAP guidelines for primary care pediatricians is “monitoring for infants who gain excessive weight.” [3, 4] They do not provide guidance on what constitutes “excessive”, but extensive evidence suggests infant weight (WT) gain greater than +0.67 Z-scores is associated with increased risk for obesity [5–7].

Given this association, one might assume that infants with rapid WT gain are gaining greater fat mass per kg increase in WT, but evidence on this point is lacking. In part, this is because most studies have only considered obesity based on body mass index (BMI) [5–7], which fails to distinguish between fat mass (FM) and fat-free mass (FFM) [8]. Knowing the proportion of WT gain due to concurrent changes in FM (and FFM) in infants with versus without rapid WT gain is important. Such knowledge may affect whether or not, or the way in which, primary care pediatricians interpret rapid WT gain during infancy as a screening tool for obesity risk.

The US CDC advise that supine length is also monitored against the WHO Standards in the first 2 years of life [2], but the role of infant linear growth in obesity etiology is often overlooked [9]. There is limited literature on the association of rapid length growth (which can also be defined as a gain greater than +0.67 Z-scores) with obesity risk and underlying changes in body composition, despite 1) the strong correlation between WT and length during infancy, and 2) the fact that BMI varies as a result of both WT and length or height [10].

Utilizing serial body composition data from air-displacement plethysmography (ADP) between 0 and 6 months and deuterium dilution (DD) between 3 and 24 months, the aim of our study was to investigate how the proportion of WT gain due to concurrent changes in FM differs between (1) infants with rapid versus normal WT gain and (2) infants with rapid versus normal length growth. We also consider “slow” versus normal comparisons and differences in the proportion of BMI change due to fat mass index (FMI) change.

## Methods

### Study

The Multicenter Infant Body Composition Reference Study (MIBCRS) was a longitudinal, prospective study of infants aged 0–24 months from six countries [11]. The inclusion criteria aligned with the WHO Multicentre Growth Reference Study (MGRS) eligibility criteria at the new-born screening to ensure minimal health, environmental, and economic constraints on growth [12]. The study complied with the International Ethical Guidelines [13], and received ethical approval from national and local ethical review committees. Written informed consent was obtained from the mothers of all infants enrolled in the study.

### Samples

The MIBCRS included two samples. In the ADP sample, 470 infants (Australia, India, South Africa) were measured within 24 h of birth, at 2 weeks, and at 1, 2, 3, 4, and 6 months. In the DD sample, 1026 infants (Brazil, Pakistan, South Africa, Sri Lanka) were measured at 3, 6, 9, 12, 15 (only South Africa), 18, and 24 months. In each sample, the first and last measurements for each infant were selected for analysis. Further, because it is known that infant WT gain, and potentially infant fat mass gain, fluctuates over these periods, we sought to ensure that the age period between the first and last measurements were fairly uniform within each sample. Therefore, in the ADP sample, infants whose first measurement was after 1 month and/or whose last measurement was before 3 months were excluded. In the DD sample, the respective cut-offs were 9 and 15 months. The resulting sample sizes were *N* = 342 (ADP) and *N* = 555 (DD). A flow chart illustrating sample selection is provided in Supplementary Fig. 1. The timings of the first and last measurements are shown in Table 1. Herein, we refer to 0–6 months (ADP) and 3–24 months (DD).

**Table 1:** Description of the two study samples.

		Air-Displacement Plethysmography (*N* = 342)	Deuterium Dilution (*N* = 555)
Sex			
Boys	*N* (%)	163 (47.7)	277 (49.9)
Girls	*N* (%)	179 (52.3)	278 (50.1)
Country			
Australia	*N* (%)	123 (29.8)	–
Brazil	*N* (%)	–	216 (38.9)
India	*N* (%)	102 (36.0)	–
Pakistan	*N* (%)	–	132 (23.8)
South Africa	*N* (%)	117 (34.2)	139 (25.1)
Sri Lanka	*N* (%)	–	68 (12.3)
WT gain			
Slow (< −0.67 Z-scores)	*N* (%)	65 (19.0)	111 (20.0)
Normal	*N* (%)	156 (45.6)	313 (56.4)
Rapid (> +0.67 Z-scores)	*N* (%)	121 (35.4)	131 (23.6)
Length growth			
Slow (< −0.67 Z-scores)	*N* (%)	52 (15.2)	108 (19.5)
Normal	*N* (%)	196 (57.3)	266 (47.9)
Rapid (> +0.67 Z-scores)	*N* (%)	94 (27.5)	181 (32.6)
Infant feeding			
Not exclusively breastfed at 3 months	*N* (%)	123 (38.8)	–
Exclusively breastfed at 3 months	*N* (%)	194 (61.2)	–
Missing	*N*	25	–
First measurement			
0 months	N (%)	325 (95.0)	–
2 weeks	N (%)	3 (0.9)	–
1 month	N (%)	14 (4.1)	–
Last measurement			
3 months	N (%)	48 (14.0)	–
4 months	N (%)	64 (18.7)	–
6 months	N (%)	230 (67.3)	–
First measurement			
3 months	N (%)	–	451 (81.3)
6 months	N (%)	–	83 (15.0)
9 months	N (%)	–	21 (3.8)
Last measurement			
15 months	N (%)	–	16 (2.9)
18 months	N (%)	–	83 (15.0)
24 months	N (%)	–	456 (82.2)
First measurement			
WT Z-score	Mean (SD)	−0.58 (0.92)	−0.29 (1.01)
Length Z-score	Mean (SD)	−0.62 (0.98)	−0.55 (1.02)
WT (kg)	Mean (SD)	3.11 (0.45)	6.28 (1.11)
FM (kg)	Mean (SD)	0.34 (0.18)	1.42 (0.65)
BMI (kg/m^2^)	Mean (SD)	12.98 (1.37)	16.85 (1.75)
FMI (kg/m^2^)	Mean (SD)	1.42 (0.70)	3.78 (1.52)
Last measurement			
WT Z-score	Mean (SD)	−0.29 (0.96)	−0.19 (1.14)
Length Z-score	Mean (SD)	−0.44 (1.02)	−0.32 (1.15)
WT (kg)	Mean (SD)	7.00 (0.84)	11.48 (1.62)
FM (kg)	Mean (SD)	1.88 (0.50)	2.50 (0.92)
BMI (kg/m^2^)	Mean (SD)	17.03 (1.73)	15.88 (1.48)
FMI (kg/m^2^)	Mean (SD)	4.57 (1.20)	3.46 (1.26)

*BMI* body mass index, *FM* fat mass, *FMI* fat mass index, *WT* weight.

### Data

Detailed measurement protocols have been described previously [11]. Briefly, at each assessment and for both samples, infant WT was measured naked, using a pediatric electronic scale (SECA 376), accurate to the nearest 5 g up to 7.5 kg and to the nearest 10 g up to 20 kg. Supine length was measured using a Harpenden stadiometer (accurate to 1 mm; Holtain Ltd) in all countries, except India and Sri Lanka, where the SECA 417 infantometer was used.

Body composition was measured for each participant on the same day as the anthropometry. In the ADP sample, PEA POD machines (Software version 3.5.0, 201, COSMED) were used following standard procedures [14]. The total body density, calculated as the ratio of WT (kg) and the measured body volume (L), was used to calculate the proportions of FM and FFM using assumed densities (0.9007 for FM, 1.063 kg/L for FFM). In the DD sample, infants received 1 g (ages 3–9 months) or 1.5 g (ages 12–24 months) of deuterium oxide (D_2_O; 99.8 atom % 2H) sterility tested. Saliva was sampled before D_2_O administration and 3 h after the administration of the dose. The enrichment of D_2_O in saliva was measured either by isotope ratio MS or by FITR using an Agilent 4500 Series spectrometer [15, 16]. Total body water was calculated using the WT of D_2_O consumed, the enrichment of the deuterium in the dose, and the enrichment of deuterium in the saliva, with a small correction (4.1%) for nonaqueous exchange of deuterium [17]. FFM was estimated by dividing by an age-related constant for the hydration of FFM [18], and FM was calculated as the difference between body WT and FFM.

In the ADP sample, at the 3-month visit, women were asked whether or not they were exclusively breastfeeding [19]. Approximately 7% of these data were missing, so all analyses involving this variable have a slightly smaller N (i.e., *N* = 317 not 342).

### Analysis

Infant WT, FM, and FFM were expressed in kg so that WT = FM + FFM. Variables were created capturing change in WT (ΔWT) and change in FM (ΔFM) between 0–6 months and 3–24 months. The percentage of ΔWT due to ΔFM was computed for each infant. Infant WT and length Z-scores were calculated according to the WHO Standards [20]. Infant WT gain and length growth were each categorized as slow (<−0.67 Z-scores), normal, or rapid (>+0.67 Z-scores) [21].

All analyses were performed for each sample separately. Descriptive statistics were produced. Paired co-ordinate arrow plots were produced to visualize the data (e.g., an arrow from FM_First_, WT_First_ to FM_last_, WT_last_ for each infant). Distributions of the percentage of ΔWT due to ΔFM were investigated using kernel density plots.

A first set of general linear models were fitted to two outcomes (ΔWT and ΔFM) using seemingly unrelated regression [22]. Models 1 (and all subsequent models) included sex, country, decimal months between first and last measurements, and first WT or FM. Models 2 included the categorical WT gain variable, models 3 included the categorical length growth variable, and models 4 (ADP sample only) included the binary exclusive breastfeeding at 3 months variable. These analyses were used to obtain estimates of mean ΔWT and ΔFM in each group (e.g., country), the percentage of ΔWT that was due to ΔFM in each group, and how those percentages differed between groups. Because the timing of the first and last measurements differed between countries (Supplementary Table 1), we performed an analysis of country differences (Models 1) restricting the sample to infants with data at 0 and 6 months (ADP sample) or 3 and 24 months (DD sample).

All analyses up to this point were repeated using BMI and FMI; both computed as kg/m^2^ so that BMI = FMI + fat-free mass index (FFMI). For the DD sample, these results are presented as supplementary material because ΔBMI between 3 and 24 months smooths over a period of increase followed by decrease, potentially producing obscure results [10].

A second set of general linear regression models were fitted directly to the percentage of ΔWT due to ΔFM variable to obtain r-squared estimates of the amount of variation explained by the independent variables (considered separately and together). Modification by sex of the effects of infant WT gain (both samples) and exclusive breastfeeding categories (ADP sample only) were examined by including two-way interactions terms, as was evidence for an a priori hypothesized interaction between infant WT gain and exclusive breastfeeding (ADP sample only). These analyses were not repeated using the percentage of ΔBMI due to ΔFMI because this variable suffered from a non-normal distribution, particularly in the DD sample.

## Results

Descriptive statistics are shown in Table 1. Approximately, 35% of infants in the ADP sample, and 24% in the DD sample, demonstrated rapid infant WT gain. Cross-tabulations of the WT gain categories against the linear growth categories are shown in Supplementary Table 2.

### ADP Sample

**Fig. 1:**
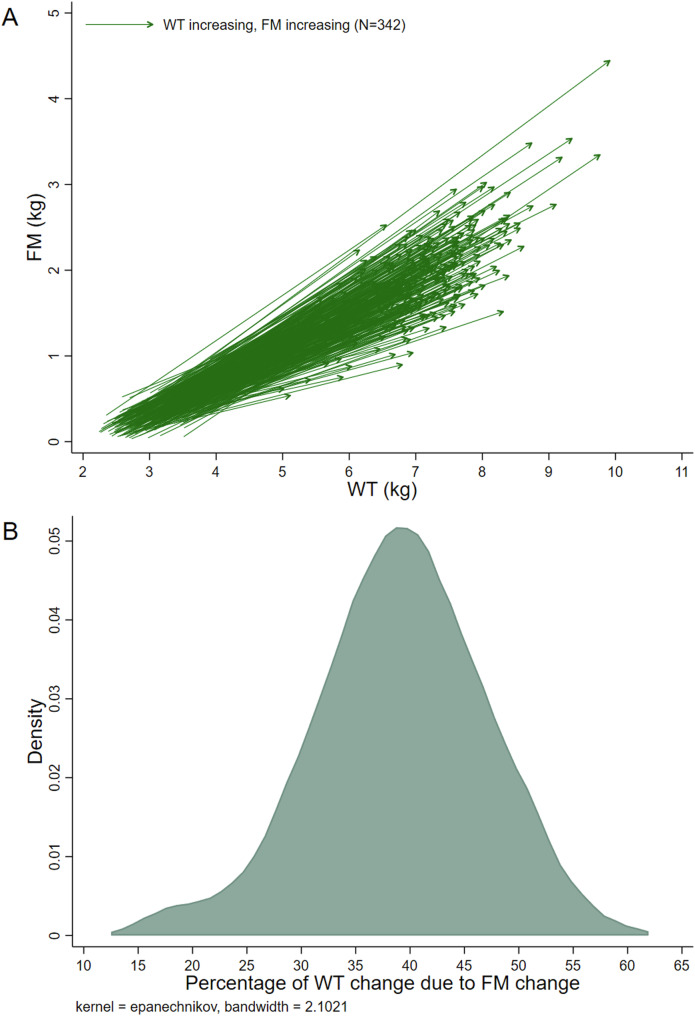
Description of fat mass change relative to weight change in the Air-Displacement Plethysmography (0–6 months) sample. **A** Paired co-ordinate arrow plot. Each line shows the data of one infant, connecting their first measurements to their last measurements (i.e., y1, x1 to y2, x2). **B** Kernel density estimate plot. Abbreviations: WT weight, FM fat mass.

Between 0 and 6 months, all infants exhibited increasing WT and FM (Fig. 1). In boys, ~37% of the average WT gain between 0 and 6 months was due to an increase in FM (Table 2). This estimate was 2.8 (1.1, 4.5) percentage points higher in girls (vs boys) and 1.8 (−0.2, 3.8) percentage points higher in infants who were exclusively breastfed at 3 months (vs those who were not). In addition, compared to the average WT gain group, this estimate was 2.9 (0.3, 5.4) percentage points lower in the slow WT gain group and 4.3 (2.9, 5.8) percentage points higher in the rapid WT gain group. Conversely, there were no statistical differences in the average fat gain as a percentage of WT gain among the length gain groups (rapid, slow, normal linear growth).

**Table 2:** The proportion of weight change due to fat mass change between first and last measurements (0–6 months) in the Air-Displacement Plethysmography sample^a^.

			Mean (kg) Estimate (95% CI)	Percentage Estimate (95% CI)	Difference Estimate (95% CI) *P*-value	
Models 1	Boys	ΔWT	4.0 (3.8, 4.1)	–		
		ΔFM	1.5 (1.4, 1.6)	37.2 (35.6, 38.8)	Referent	
	Girls	ΔWT	3.6 (3.4, 3.7)	–		
		ΔFM	1.4 (1.3, 1.5)	40.0 (38.3, 41.6)	2.8 (1.1, 4.5)	0.001
Models 1	Australia	ΔWT	4.0 (3.8, 4.1)	–		
		ΔFM	1.5 (1.4, 1.6)	37.2 (35.6, 38.8)	Referent	
	India	ΔWT	4.1 (4.0, 4.3)	–		
		ΔFM	1.6 (1.5, 1.7)	38.7 (37.0, 40.3)	1.5 (−0.5, 3.5)	0.150
	South Africa	ΔWT	4.2 (4.1, 4.4)	–		
		ΔFM	1.6 (1.5, 1.7)	38.2 (36.6, 39.8)	1.0 (−0.9, 2.9)	0.305
Models 2	Slow WT gain (<−0.67 Z-scores)	ΔWT	3.1 (3.0, 3.3)	–		
		ΔFM	1.1 (1.0, 1.2)	34.7 (32.2, 37.3)	−2.9 (−5.4, −0.3)	0.027
	Normal	ΔWT	4.0 (3.9, 4.1)	–		
		ΔFM	1.5 (1.4, 1.6)	37.6 (35.9, 39.3)	Referent	
	Rapid WT gain (>+0.67 Z-scores)	ΔWT	4.9 (4.8, 5.0)	–		
		ΔFM	2.1 (1.9, 2.2)	42.0 (40.4, 43.5)	4.3 (2.9, 5.8)	<0.001
Models 3^b^	Slow length growth (<−0.67 Z-scores)	ΔWT	3.7 (3.5, 3.9)	–		
		ΔFM	1.3 (1.2, 1.5)	36.6 (34.1, 39.1)	−1.0 (−3.5, 1.5)	0.442
	Normal	ΔWT	4.0 (3.9, 4.2)	–		
		ΔFM	1.5 (1.4, 1.6)	37.6 (35.9, 39.3)	Referent	
	Rapid length growth (>+0.67 Z-scores)	ΔWT	4.4 (4.2, 4.6)	–		
		ΔFM	1.6 (1.5, 1.8)	37.3 (35.2, 39.5)	−0.3 (−2.0, 1.5)	0.779
Models 4	Not exclusively breastfed at 3 months	ΔWT	4.1 (3.9, 4.3)	–		
		ΔFM	1.5 (1.3, 1.6)	35.9 (33.8, 38.0)	Referent	
	Exclusively breastfed at 3 months	ΔWT	3.9 (3.7, 4.0)	–		
		ΔFM	1.5 (1.4, 1.6)	37.7 (36.0, 39.5)	1.8 (−0.2, 3.8)	0.077

*FM* fat mass, *WT* weight.

^a^Estimates are from seemingly unrelated regression models (outcomes = ΔWT and FM Change) adjusted for sex (boys [referent], girls), country (Australia [referent], India, South Africa), decimal months between the first and last measurements (centered at the mean), and WT or FM at the first measurement (centered at the mean).

**Fig. 2:**
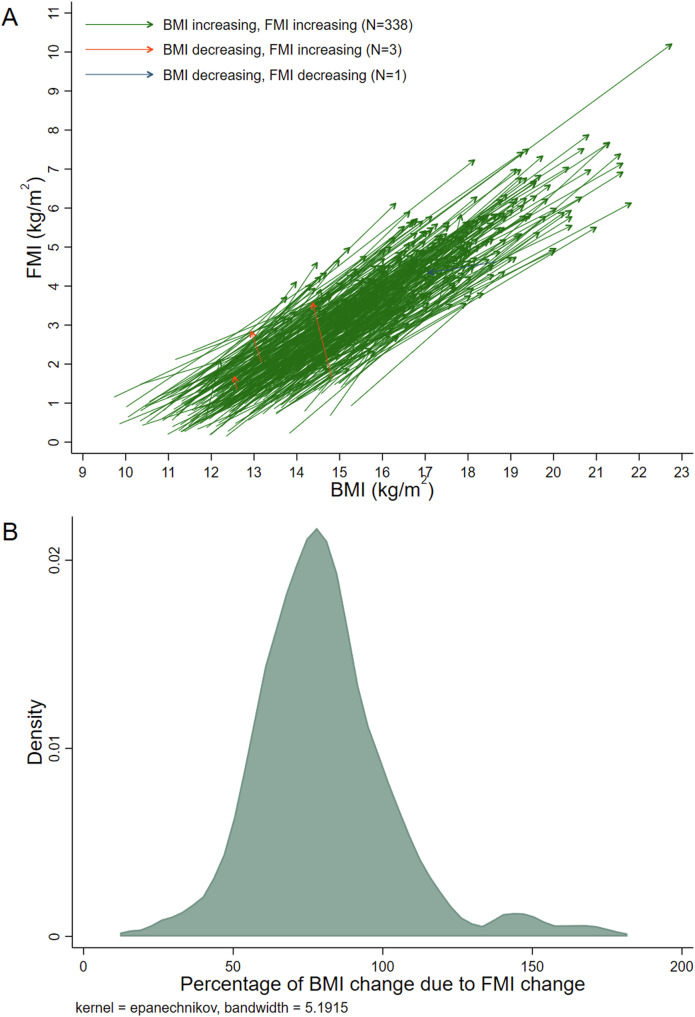
Description of FMI change relative to BMI change in the Air-Displacement Plethysmography (0–6 months) sample. **A** Paired co-ordinate arrow plot. Each line shows the data of one infant, connecting their first measurements to their last measurements (i.e., y1, x1 to y2, x2). **B** Kernel density estimate plot. Excludes 10 infants with extreme values (<10% or >180%). Abbreviations: BMI body mass index, FMI fat mass index.

The distribution of the percentage of ΔBMI due to ΔFMI was slightly skewed and leptokurtic (Fig. 2). The percentage was estimated to be consistently high, ranging between 66 and 81% (Table 3). Again, the estimate was higher in girls (vs boys) and in infants who were exclusively breastfed at 3 months (vs those who were not). However, estimates of differences between the infant WT gain groups were in the opposite direction to those observed in Table 2, with the slow WT gain group demonstrating the highest proportion of ΔBMI due to ΔFMI.

**Table 3:** The proportion of BMI change due to FMI change between first and last measurements (0–6 months) in the Air-Displacement Plethysmography sample^a^.

			Mean (kg/m^2^) Estimate (95% CI)	Percentage Estimate (95% CI)	Difference Estimate (95% CI) *P*-value	
Models 1	Boys	ΔBMI	4.0 (3.7, 4.4)	–		
		ΔFMI	2.9 (2.7, 3.2)	72.3 (68.8, 75.7)	Referent	
	Girls	ΔBMI	3.8 (3.4, 4.1)	–		
		ΔFMI	3.0 (2.8, 3.2)	80.1 (76.3, 83.9)	7.9 (4.1, 11.6)	<0.001
Models 1	Australia	ΔBMI	4.0 (3.7, 4.4)	–		
		ΔFMI	2.9 (2.7, 3.2)	72.3 (68.8, 75.7)	Referent	
	India	ΔBMI	4.0 (3.6, 4.4)	–		
		ΔFMI	3.1 (2.8, 3.4)	77.8 (73.7, 81.9)	5.5 (0.6, 10.5)	0.029
	South Africa	ΔBMI	4.5 (4.2, 4.9)	–		
		ΔFMI	3.3 (3.0, 3.6)	72.5 (69.3, 75.8)	0.3 (−3.7, 4.2)	0.900
Models 2	Slow WT gain (<−0.67 Z-scores)	ΔBMI	2.6 (2.3, 2.9)	–		
		ΔFMI	2.1 (1.9, 2.4)	81.2 (73.4, 89.0)	8.4 (1.1, 15.7)	0.024
	Normal	ΔBMI	4.0 (3.7, 4.3)	–		
		ΔFMI	2.9 (2.7, 3.1)	72.8 (68.8, 76.8)	Referent	
	Rapid WT gain (>+0.67 Z-scores)	ΔBMI	6.0 (5.6, 6.3)	–		
		ΔFMI	4.2 (4.0, 4.5)	70.9 (67.8, 74.0)	−1.9 (−5.2, 1.4)	0.267
Models 3^b^	Slow length growth (<−0.67 Z-scores)	ΔBMI	4.3 (3.8, 4.8)	–		
		ΔFMI	2.8 (2.5, 3.2)	66.4 (62.0, 70.9)	−7.8 (−12.5, −3.1)	0.001
	Normal	ΔBMI	4.0 (3.6, 4.3)	–		
		ΔFMI	3.0 (2.7, 3.2)	74.2 (70.4, 78.0)	Referent	
	Rapid length growth (> +0.67 Z-scores)	ΔBMI	3.7 (3.2, 4.2)	–		
		ΔFMI	2.9 (2.6, 3.3)	80.1 (74.2, 86.0)	5.9 (1.2, 10.5)	0.014
Models 4	Not exclusively breastfed at 3 months	ΔBMI	4.3 (3.9, 4.8)	–		
		ΔFMI	2.9 (2.6, 3.2)	67.4 (63.3, 71.4)	Referent	
	Exclusively breastfed at 3 months	ΔBMI	3.9 (3.5, 4.2)	–		
		ΔFMI	2.9 (2.6, 3.1)	74.6 (70.7, 78.4)	7.2 (3.1, 11.3)	0.001

*BMI* body mass index, *FMI* fat mass index, *WT* weight.

^a^Estimates are from seemingly unrelated regression models (outcomes = ΔBMI and FMI Change) adjusted for sex (boys [referent], girls), country (Australia [referent], India, South Africa), decimal months between the first and last measurements (centered at the mean), and BMI or FMI at the first measurement (centered at the mean).

In the analyses that restricted the sample to infants with data at 0 and 6 months (Supplementary Tables 3 and 4), evidence was found that the percentage of ΔWT due to ΔFM (and the percentage of ΔBMI due to ΔFMI) was higher in India (but not in South Africa) than Australia.

### DD Sample

**Fig. 3:**
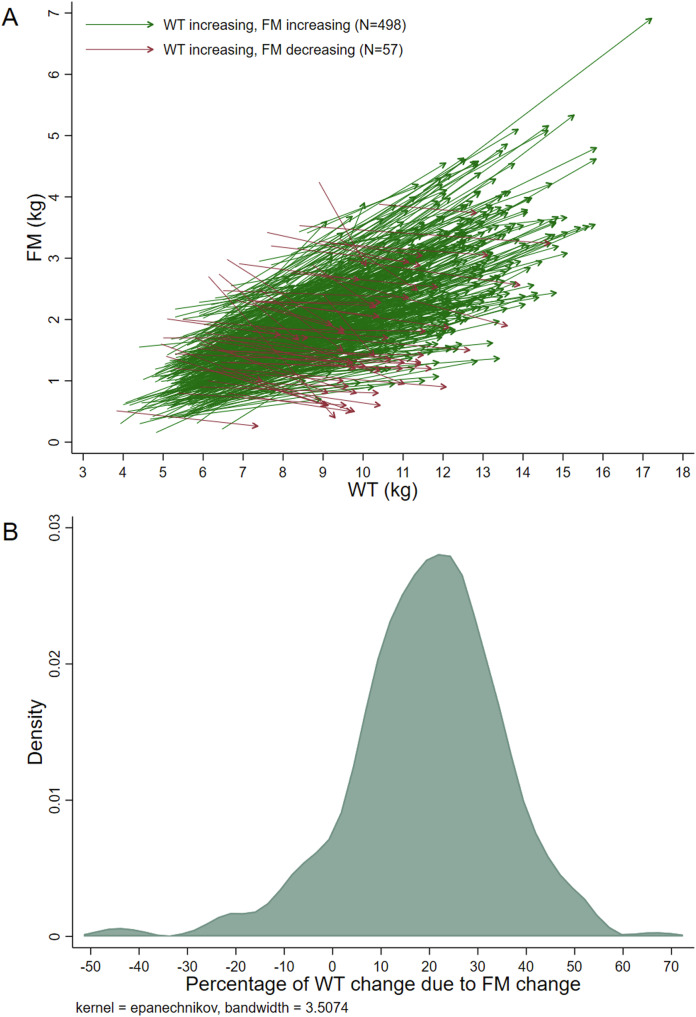
Description of fat mass change relative to weight change in the Deuterium Dilution (3–24 months) sample. **A** Paired co-ordinate arrow plot. Each line shows the data of one infant, connecting their first measurements to their last measurements (i.e., y1, x1 to y2, x2). **B** Kernel density estimate plot. Excludes 5 infants with extreme values (<−50% or >70%). Abbreviations: WT weight, FM fat mass.

Between 3 and 24 months, 90% of infants demonstrated increasing WT and FM and 10% demonstrated increasing WT yet decreasing FM (Fig. 3). Descriptive statistics for each group are shown in Supplementary Table 5. Despite only 12.3% of the DD sample being Sri Lankan, 40% of the infants in the decreasing FM group were Sri Lankan. Approximately, 19% of the average ΔWT was due to a change in ΔFM (Table 4), again with this estimate being higher in girls (vs boys) and in infants with rapid (vs normal) WT gain. Also like the findings in the ADP sample, differences between the infant linear growth groups were much smaller than those observed between the WT gain groups. Unlike the results for the ADP sample, however, there were more notable differences between countries. For example, while 19.0% (17.3, 20.7) of the average ΔWT was due to ΔFM in infants from Brazil, this estimate was 5.9% (2.0, 9.8) for infants from Sri Lanka. This, and other, country differences were still present after restricting the sample to infants with data at 3 and 24 months (Supplementary Table 6).

**Table 4:** The proportion of weight change due to fat mass change between first and last measurements (3–24 months) in the Deuterium Dilution sample^a^.

			Mean (kg) Estimate (95% CI)	Percentage Estimate (95% CI)	Difference Estimate (95% CI) *P*-value	
Models 1	Boys	ΔWT	5.9 (5.7, 6.1)	–		
		ΔFM	1.1 (1.0, 1.2)	19.0 (17.3, 20.7)	Referent	
	Girls	ΔWT	5.7 (5.5, 5.9)	–		
		ΔFM	1.3 (1.1, 1.4)	21.9 (20.2, 23.6)	2.8 (1.0, 4.7)	0.002
Models 1	Brazil	ΔWT	5.9 (5.7, 6.1)	–		
		ΔFM	1.1 (1.0, 1.2)	19.0 (17.3, 20.7)	Referent	
	Pakistan	ΔWT	5.0 (4.7, 5.2)	–		
		ΔFM	0.7 (0.6, 0.9)	14.3 (11.7, 16.9)	−4.7 (−7.4, −2.0)	0.001
	South Africa	ΔWT	5.0 (4.7, 5.2)	–		
		ΔFM	1.5 (1.3, 1.6)	29.6 (27.1, 32.0)	10.6 (7.9, 13.3)	<0.001
	Sri Lanka	ΔWT	4.6 (4.3, 4.9)	–		
		ΔFM	0.3 (0.1, 0.5)	5.9 (2.0, 9.8)	−13.2 (−17.1, −9.2)	<0.001
Models 2	Slow WT gain (<−0.67 Z-scores)	ΔWT	4.3 (4.0, 4.5)	–		
		ΔFM	0.6 (0.4, 0.8)	14.3 (10.5, 18.1)	−4.1 (−7.5, −0.8)	0.015
	Normal	ΔWT	5.5 (5.3, 5.6)	–		
		ΔFM	1.0 (0.9, 1.1)	18.4 (16.4, 20.4)	Referent	
	Rapid WT gain (>+0.67 Z-scores)	ΔWT	7.0 (6.8, 7.2)	–		
		ΔFM	1.5 (1.4, 1.7)	21.9 (20.0, 23.8)	3.5 (1.4, 5.6)	0.001
Models 3^b^	Slow length growth (<−0.67 Z-scores)	ΔWT	5.0 (4.7, 5.3)	–		
		ΔFM	0.8 (0.6, 1.0)	16.3 (12.9, 19.7)	−2.5 (−5.4, 0.4)	0.094
	Normal	ΔWT	5.5 (5.3, 5.7)	–		
		ΔFM	1.0 (0.9, 1.2)	18.8 (16.7, 21.0)	Referent	
	Rapid length growth (>+0.67 Z-scores)	ΔWT	6.4 (6.2, 6.6)	–		
		ΔFM	1.2 (1.1, 1.4)	19.6 (17.8, 21.5)	0.8 (−1.3, 2.9)	0.454

*FM* fat mass, *WT* weight.

^a^Estimates are from seemingly unrelated regression models (outcomes = ΔWT and FM Change) adjusted for sex (boys [referent], girls), country (Brazil [referent], Pakistan, South Africa, Sri Lanka), decimal months between the first and last measurements (centered at the mean), and WT or FM at the first measurement (centered at the mean).

Approximately 50% of infants had decreasing BMI and FMI between 3 and 24 months, while the remaining infants demonstrated one of the other combinations (Supplementary Fig. 2). The descriptive statistics for each of the four groups are shown in Supplementary Table 7. And the estimates from the first set of regression models are shown in Supplementary Tables 8, 9.

### Variance explained

Using the second set of regression models (which included only infants with data at 0 and 6 mo, and 3 and 24 mo), infant WT gain explained the most variance (11.8%) in the outcome (percentage of ΔWT due to ΔFM) in the ADP sample (Table 5). Conversely, country explained the most variance (8.2%) in the DD sample.

**Table 5:** The variation in the percentage of weight change due to fat mass change explained by each independent variable considered separately and all independent variables considered together^a^.

	Air-Displacement Plethysmography sample (0–6 months)		Deuterium Dilution sample (3–24 months)	
	Percentage of ΔWT due to ΔFM		Percentage of ΔWT due to ΔFM^b^	
	*R*^2^(100)	Adj *R*^2^(100)	*R*^2^(100)	Adj *R*^2^(100)
Sex	1.8	1.5	1.0	0.8
Country	1.0	0.5	8.2	7.7
WT gain (categories)	11.8	11.3	4.1	3.7
Length growth (categories)	0.4	−0.2	0.7	0.4
Exclusively breastfed at 3 months	0.02	−0.3	–	–
All variables (excluding breastfeeding)	17.0	15.3	14.2	12.9
All variables (including breastfeeding)	21.2	19.2	–	–

*FM* fat mass, *WT* weight.

^a^Estimates are from regression models (outcome = percentage of ΔWT due to ΔFM).

^b^Excludes 5 infants with extreme outcome values (<−50% or >70%).

### Interactions

The association of slow (vs normal) WT gain with a lower percentage of ΔWT due to ΔFM was attenuated to the null among infants who were exclusively breastfed at 3 months in the ADP sample (Supplementary Table 10, Supplementary Fig. 3). Likelihood ratio tests comparing models with vs without interaction terms were however null (*p*-value 0.068).

## Discussion

Compared to other periods of development, infancy is a period characterized by particularly rapid WT gain; however, proper interpretation of WT gain or WT loss depend on assumptions regarding tissue-specific dynamics, including the relative gain or loss of lean mass and adipose tissue mass. For example, it is widely assumed that infants who grow quickly are accruing relatively greater adipose tissue than those who are growing more slowly, which then leads to the increased risk of obesity in childhood [6, 7, 9]. However, sufficiently large datasets having longitudinal infant body composition information to objectively test this assumption have been lacking, with most studies having recruited a small number of participants from a single study site and in higher income settings. The studies that do exist have typically been used to produce growth charts and/or investigate the correlates of infant body composition [23–28]. The present study has a different focus and provides novel data on the proportion of ΔWT due to ΔFM. The key finding is that, between 0–6 and 3–24 months, the proportion of ΔWT due to ΔFM was higher (and consequently the proportion of ΔWT due to ΔFFM was lower) in infants with rapid versus normal WT gain, but not in infants with rapid versus normal linear growth.

It is well known that rapid infant WT gain is associated with childhood obesity, but most of this literature has defined the outcome using BMI [5–7]. In the systematic review and metanalysis of Zheng et al., for example, 11 of the 17 included studies only considered “adiposity” based on BMI [7]. In addition to the well-known limitations of BMI in childhood [8, 29, 30], the association of rapid infant WT gain with child BMI is partly self-fulfilling [31]. A group with rapid infant WT gain between say 0–1 years will inevitably have higher WT (and BMI) at 1 year than a group without rapid WT gain, and because WT (and BMI) tracks with age they will also have higher WT (and BMI) in childhood. The number of papers that have considered true “adiposity” outcomes from, for example, dual-energy x-ray absorptiometry is actually very limited; four in the Zheng et al. review [7]. Appropriate adjustment for total body size in these papers is crucial because, on average, heavier children will have a higher absolute FM [32]. The problem is that many papers mistakenly adjust for body WT when they should adjust for body height; three of the four papers with true “adiposity” outcomes in the Zheng review misguidedly considered percent body fat [33, 34]. Our paper circumvented some of the challenges and limitations found in the rapid infant WT gain and childhood obesity literature by investigating concurrent changes in adiposity in infants with different types of growth. In addition, the study samples are from geographically diverse areas and include infants from lower-middle, upper-middle, and high income countries which adds to the generalizability of the results.

In addition to the proportion of ΔWT due to ΔFM being higher in infants with rapid versus normal WT gain, it was lower in infants with slow versus normal WT gain. The relationship between infant WT gain and the amount of fat gained per kg increase in body WT might not, however, be linear. Between 0 and 6 months, the ΔWT due to ΔFM effect size for slow versus normal (−2.9) was weaker than that for rapid versus normal (+ 4.3). This is in agreement with previous research reporting a non-linear relationship between infant WT gain and adolescent BMI, such that the association was weaker at the lower end of the exposure distribution [35].

While the limitations of using BMI during infancy and early childhood are well known [8, 29, 30], we did find that mean BMI change between 0 and 6 months largely reflected an increase in mean adiposity. This makes sense given knowledge (1) that the peaks in infant BMI and FM coincide at about 6–9 months of age and (2) that infant BMI is correlated with FM, even more so than are WT-for-length Z-scores [10, 36–38]. Building on this cross-sectional evidence, serial measurement of BMI at 0 and 6 months may be a justified approach for studying differences between groups of infants in early-life fat accumulation. This does not, however, necessarily mean that BMI change can be used to accurately assess the fat accumulation of an individual infant [39]. Our data show, for example, that the changes in FMI as a proportion of BMI change varied greatly between individual infants, ranging from less than 20% to over 100%. Further, between 3 and 24 months, BMI change is likely to be a very poor indicator of changes in fat mass, with highly variable (e.g., between sexes) predictive ability.

The dynamics of early life growth are complex and different for the two cohorts in our study. Infants are born with very little body fat, making them vulnerable to the environment and insults that may draw on energy reserves [11, 36]. Subsequently, infant WT gain is proportionally faster than linear growth, and BMI and adiposity peak at ~6–9 months [10]. The measurements in the ADP sample align well with this timescale, and it was no surprise that infant WT gain (categories) explained the most variance in the percentage of ΔWT due to ΔFM between 0 and 6 months. Conversely, the measurements in the DD sample capture change from before the peak in adiposity (3 months) to toddlerhood (24 months). Changes in WT and body composition in this period are influenced by many more factors, including the transition from a liquid diet of human milk or formula to a full array of complementary solid food, increasing motor ability, and exposure to environmental pathogens and developing immune systems [36]. Because these environmental factors differ between countries, it seems intuitive that country of origin explained the most variance in the percentage of ΔWT due to ΔFM between 3 and 24 months. Compared to Brazil, South Africa had a higher percentage of ΔWT due to ΔFM, while the two South Asian countries (Pakistan and Sri Lanka) had a lower percentage. This makes sense given knowledge of the high rates of pediatric obesity in South Africa and wasting in South Asia [40–42], and previously published country differences in growth and body composition in the MIBCRS [43].

There are two main strengths of this paper. First, the serial assessment of body composition using ADP and DD in a large and diverse population sample. Second, inclusion criteria that aligned with the WHO MGRS at recruitment, which means the results can be interpreted to be representative of generally “healthy” growing infants [12]. In terms of limitations, due to attrition and missing data, not all infants were measured at 0 and 6 months or 3 and 24 months. The ADP sample comprised 73% of all infants in that cohort, while the DD sample comprised 54% of all infants in that cohort [11]. Differential selection into our sample could have biased results, although the homogenous nature of the samples (due to the strict inclusion criteria) might go some way towards limiting any potential bias [44]. Because of the sample sizes, we were not able to investigate body composition changes in infants with more extreme WT gain or linear growth. Of course, there will be a correlation between infant WT gain and linear growth, but not all infants with rapid infant WT gain will demonstrate rapid linear growth. In the ADP sample, between 0 and 6 months, nearly 60% of infants with rapid WT gain did not demonstrate rapid linear growth. This is the group likely to have the highest obesity risk, and perhaps amount of fat gain per kg increase in body WT, but unfortunately our sample sizes were not large enough to define groups according to both WT gain and length growth categories. There are also likely some infants in whom rapid WT gain is beneficial because their starting point (e.g., at birth) is characterized by insufficient adipose tissue. Further research with much more specific groups (e.g., term birth, small-for-gestational age, breastfed, with rapid linear growth) is needed to understand which infants may actually benefit from rapid WT gain.

## Conclusion

Pediatricians should monitor growth with the understanding that on average, infants crossing upward through the WT centiles are not just getting heavier, they are putting on more FM per kg increase in WT. This does not mean that all infants with rapid WT gain are putting on too much fat. Indeed, in some infants, rapid WT gain and accompanying increases in adiposity may actually be a good thing (and not lead to obesity). Conversely, on average, infants with versus without rapid length growth gain proportionally the same amount of FM and FFM.

## Supplementary information

Supplementary Material

## Data Availability

Data described in the manuscript, code book, and analytic code will be made available upon request to WJ.
