# Supplementary material for: The proportion of weight gain due to change in fat mass in infants with vs without rapid growth
Source: Eur J Clin Nutr. 2024 Nov 5;79(3):237–48. doi: 10.1038/s41430-024-01534-5 (PMC11893438; doi:10.1038/s41430-024-01534-5)

**Supplementary Material**

**The proportion of weight gain due to change in fat mass in infants with vs without rapid growth**

William Johnson, Lukhanyo H Nyati, Shabina Ariff, Tanvir Ahmad, Nuala M Byrne, Leila I Cheikh Ismail, Caroline S Costa, Ellen W Demerath, Divya J Priscilla, Andrew P Hills, Rebecca Kuriyan, Anura V Kurpad, Cornelia U Loechl, M Nishani Lucas, Ina S Santos, Christine Slater, V Pujitha Wickramasinghe, Shane A Norris, Alexia J Murphy-Alford, and on behalf of the Multicenter Infant Body Composition Reference Study (MIBCRS)

The Multicenter Infant Body Composition Reference Study (MIBCRS) group

Renata M Bielemann^1^, Caroline S Costa^1^, Ina S Santos^1^, Neiva CJ Valle^1^, Andrew P Hills^2^, Nuala M Byrne^2^, Anne Hanley^2^, Manoja P Herath^2^, Steven J Street^2^, Kiran DK Ahuja^2^, Jeffrey M Beckett^2^, Sisitha Jayasinghe^2^, Shabina Ariff^3^, Laila Charania^3^, Sajid Soofi^3^, V Pujitha Wickramasinghe^4^, Nishani Lucas^4^, Upul Senerath^4^, Pulani Lanerolle^4^, Lukhanyo Nyati^5^, Alexia J Murphy-Alford^6^, Cornelia U Loechl^6^, Najat Moktar^6^, Christine Slater^6^, Rebecca Kuriyan^7^, Michele P Christian^7^, Priscilla J Divya^7^, Anura V Kurpad^7^, Tanvir Ahmad^8^, Ayesha Yameen^8^, Leila Cheikh Ismail^9,10^, Shane A Norris^5, 11^

^1^Federal University of Pelotas, Brazil

^2^University of Tasmania, Australia

^3^The Aga Khan University, Pakistan

^4^University of Colombo, Sri Lanka

^5^University of the Witwatersrand, South Africa

^6^International Atomic Energy Agency, Austria

^7^St John’s Research Institute, India

^8^Isotope Application Division, Pakistan Institute of Nuclear Science and Technology (PINSTECH), Pakistan

^9^University of Sharjah, United Arab Emirates

^10^University of Oxford, UK

^11^University of Southampton, UK

Supplementary Table 1. The timing of first and last measurements, stratified by country

|  |  | Australia | Brazil | India | Pakistan | South Africa | Sri Lanka |
| --- | --- | --- | --- | --- | --- | --- | --- |
| Air-Displacement Plethysmography sample (0-6 months) | First measurement |  |  |  |  |  |  |
|  | 0 months | 119 (96.8) | -- | 100 (98.0) | -- | 106 (90.6) | -- |
|  | 2 weeks | 2 (1.6) | -- | 1 (1.0) | -- |  | -- |
|  | 1 month | 2 (1.6) | -- | 1 (1.0) | -- | 11 (9.4) | -- |
|  | Last measurement |  |  |  |  |  |  |
|  | 3 months | 20 (16.3) | -- | 3 (2.9) | -- | 25 (21.4) | -- |
|  | 4 months | 18 (14.6) | -- | 3 (2.9) | -- | 43 (36.8) | -- |
|  | 6 months | 85 (69.1) | -- | 96 (94.1) | -- | 49 (41.9) | -- |
| Deuterium Dilution sample (3-24 months) | First measurement |  |  |  |  |  |  |
|  | 3 months | -- | 172 (79.6) | -- | 118 (89.4) | 102 (73.4) | 59 (86.8) |
|  | 6 months | -- | 35 (16.2) | -- | 13 (9.9) | 28 (20.1) | 7 (10.3) |
|  | 9 months | -- | 9 (4.2) | -- | 1 (0.8) | 9 (6.5) | 2 (2.9) |
|  | Last measurement |  |  |  |  |  |  |
|  | 15 months | -- |  | -- |  | 16 (11.5) |  |
|  | 18 months | -- | 25 (11.6) | -- | 22 (16.7) | 13 (9.4) | 23 (33.8) |
|  | 24 months | -- | 191 (88.4) | -- | 110 (83.3) | 110 (79.1) | 45 (66.2) |

Supplementary Table 2. Cross-tabulations of weight gain categories against linear growth categories, with column percentages

|  |  | Air-Displacement Plethysmography (0-6 months) | | | Deuterium Dilution sample (3-24 months) | | |
| --- | --- | --- | --- | --- | --- | --- | --- |
|  |  | WT gain | | | WT gain | | |
|  |  | Slow (N=65) | Normal (N=156) | Rapid (N=121) | Slow (N=111) | Normal (N=313) | Rapid (N=131) |
| Length growth |  |  |  |  |  |  |  |
| Slow (< -0.67 Z-scores) | N (%) | 25 (38.5) | 19 (22.2) | 8 (6.6) | 53 (47.8) | 52 (16.6) | 3 (2.3) |
| Normal | N (%) | 36 (55.4) | 99 (63.5) | 61 (50.4) | 50 (45.1) | 174 (55.6) | 42 (32.1) |
| Rapid (> +0.67 Z-scores) | N (%) | 4 (6.2) | 38 (24.4) | 52 (43.0) | 8 (7.2) | 87 (27.8) | 86 (65.7) |

Abbreviations: weight (WT)

Supplementary Table 3. The proportion of weight change due to fat mass change in the Air-Displacement Plethysmography sample: analysis of country differences restricting the sample to infants whose first measurement was at 0 months and whose last measurement was at 6 months^a^

|  | Full sample  N | Restricted sample  N |  | Mean (kg)  Estimate (95% CI) | Percentage  Estimate (95% CI) | Difference  Estimate (95% CI) P-value | |
| --- | --- | --- | --- | --- | --- | --- | --- |
| Australia | 123 | 82 | ΔWT | 4.3 (4.1, 4.5) | -- |  |  |
|  |  |  | ΔFM | 1.5 (1.4, 1.6) | 35.3 (33.4, 37.3) | Referent |  |
| India | 102 | 94 | ΔWT | 4.5 (4.3, 4.7) | -- |  |  |
|  |  |  | ΔFM | 1.7 (1.6, 1.8) | 37.9 (36.3, 39.6) | 2.6 (0.3, 4.8) | 0.026 |
| South Africa | 117 | 47 | ΔWT | 4.5 (4.3, 4.7) | -- |  |  |
|  |  |  | ΔFM | 1.7 (1.5, 1.8) | 36.6 (34.4, 38.7) | 1.2 (-1.3, 3.7) | 0.338 |

Abbreviations: fat mass (FM), weight (WT)

^a^Estimates are from seemingly unrelated regression models (outcomes = ΔWT and ΔFM) adjusted for sex (boys [referent], girls), country (Australia [referent], India, South Africa), decimal months between the first and last measurements (centered at the mean), and WT or FM at the first measurement (centered at the mean).

Supplementary Table 4. The proportion of BMI change due to FMI change in the Air-Displacement Plethysmography sample: analysis of country differences restricting the sample to infants whose first measurement was at 0 months and whose last measurement was at 6 months^a^

|  | Full sample  N | Restricted sample  N |  | Mean (kg/m^2^)  Estimate (95% CI) | Percentage  Estimate (95% CI) | Difference  Estimate (95% CI) P-value | |
| --- | --- | --- | --- | --- | --- | --- | --- |
| Australia | 123 | 82 | ΔBMI | 4.0 (3.6, 4.4) | -- |  |  |
|  |  |  | ΔFMI | 2.8 (2.6, 3.1) | 70.8 (66.4, 75.1) | Referent |  |
| India | 102 | 94 | ΔBMI | 4.0 (3.7, 4.4) | -- |  |  |
|  |  |  | ΔFMI | 3.2 (2.9, 3.4) | 78.5 (74.0, 82.9) | 7.7 (1.8, 13.6) | 0.011 |
| South Africa | 117 | 47 | ΔBMI | 4.5 (4.0, 5.0) | -- |  |  |
|  |  |  | ΔFMI | 3.2 (2.9, 3.6) | 72.1 (67.3, 76.8) | 1.3 (-4.1, 6.8) | 0.640 |

Abbreviations: body mass index (BMI), fat mass index (FMI), weight (WT)

^a^Estimates are from seemingly unrelated regression models (outcomes = ΔBMI and ΔFMI) adjusted for sex (boys [referent], girls), country (Australia [referent], India, South Africa), decimal months between the first and last measurements (centered at the mean), and BMI or FMI at the first measurement (centered at the mean).

Supplementary Table 5. Descriptive statistics for the two groups of infants shown in Figure 3, Panel A

|  |  | Deuterium Dilution sample (3-24 months) | | |
| --- | --- | --- | --- | --- |
|  |  | Total  (N=555) | WT increasing,  FM increasing  (N=498, 89.7%) | WT increasing,  FM decreasing  (N=57, 10.3%) |
| Sex |  |  |  |  |
| Boys | N (%) | 277 (49.9) | 243 (48.8) | 34 (59.7) |
| Girls | N (%) | 278 (50.1) | 255 (51.2) | 23 (40.4) |
| Country |  |  |  |  |
| Brazil | N (%) | 216 (38.9) | 210 (42.2) | 6 (10.5) |
| Pakistan | N (%) | 132 (23.8) | 122 (24.5) | 10 (17.5) |
| South Africa | N (%) | 139 (25.1) | 121 (24.3) | 18 (31.6) |
| Sri Lanka | N (%) | 68 (12.3) | 45 (9.0) | 23 (40.4) |
| WT gain |  |  |  |  |
| Slow (< -0.67 Z-scores) | N (%) | 111 (20.0) | 85 (17.1) | 26 (45.6) |
| Normal | N (%) | 313 (56.4) | 285 (57.2) | 28 (49.1) |
| Rapid (> +0.67 Z-scores) | N (%) | 131 (23.6) | 128 (25.7) | 3 (5.3) |
| Length growth |  |  |  |  |
| Slow (< -0.67 Z-scores) | N (%) | 108 (19.5) | 90 (18.1) | 18 (31.5) |
| Normal | N (%) | 266 (47.9) | 236 (47.4) | 30 (52.6) |
| Rapid (> +0.67 Z-scores) | N (%) | 181 (32.6) | 172 (34.5) | 9 (15.8) |
| First measurement |  |  |  |  |
| 3 months | N (%) | 451 (81.3) | 419 (84.1) | 32 (56.1) |
| 6 months | N (%) | 83 (15.0) | 64 (12.9) | 19 (33.3) |
| 9 months | N (%) | 21 (3.8) | 15 (3.0) | 6 (10.5) |
| Last measurement |  |  |  |  |
| 15 months | N (%) | 16 (2.9) | 15 (3.0) | 1 (1.8) |
| 18 months | N (%) | 83 (15.0) | 61 (12.3) | 22 (38.6) |
| 24 months | N (%) | 456 (82.2) | 422 (84.7) | 34 (59.7) |
| First measurement |  |  |  |  |
| WT Z-score | Mean (SD) | -0.29 (1.01) | -0.31 (1.01) | -0.19 (1.03) |
| Length Z-score | Mean (SD) | -0.55 (1.02) | -0.54 (1.01) | -0.59 (1.07) |
| WT (kg) | Mean (SD) | 6.28 (1.11) | 6.21 (1.05) | 6.92 (1.36) |
| FM (kg) | Mean (SD) | 1.42 (0.65) | 1.35 (0.58) | 2.05 (0.86) |
| BMI (kg/m^2^) | Mean (SD) | 16.85 (1.75) | 16.79 (1.72) | 17.33 (1.91) |
| FMI (kg/m^2^) | Mean (SD) | 3.78 (1.52) | 3.63 (1.39) | 5.12 (1.93) |
| Last measurement |  |  |  |  |
| WT Z-score | Mean (SD) | -0.19 (1.14) | -0.12 (1.12) | -0.81 (1.07) |
| Length Z-score | Mean (SD) | -0.32 (1.15) | -0.26 (1.15) | -0.87 (1.02) |
| WT (kg) | Mean (SD) | 11.48 (1.62) | 11.59 (1.60) | 10.50 (1.50) |
| FM (kg) | Mean (SD) | 2.50 (0.92) | 2.60 (0.87) | 1.60 (0.83) |
| BMI (kg/m^2^) | Mean (SD) | 15.88 (1.48) | 15.93 (1.49) | 15.42 (1.29) |
| FMI (kg/m^2^) | Mean (SD) | 3.46 (1.26) | 3.59 (1.20) | 2.33 (1.16) |

Abbreviations: body mass index (BMI), fat mass (FM), fat mass index (FMI), weight (WT)

Supplementary Table 6. The proportion of weight change due to fat mass change in the Deuterium Dilution sample: analysis of country differences restricting the sample to infants whose first measurement was at 3 months and whose last measurement was at 24 months^a^

|  | Full sample  N | Restricted sample  N |  |  | Mean (kg)  Estimate (95% CI) | Percentage  Estimate (95% CI) | Difference  Estimate (95% CI) P-value | |
| --- | --- | --- | --- | --- | --- | --- | --- | --- |
| Brazil | 216 | 150 |  | ΔWT | 6.4 (6.1, 6.6) | -- |  |  |
|  |  |  |  | ΔFM | 1.3 (1.2, 1.5) | 20.7 (18.9, 22.6) | Referent |  |
| Pakistan | 132 | 99 |  | ΔWT | 5.5 (5.3, 5.8) | -- |  |  |
|  |  |  |  | ΔFM | 1.0 (0.9, 1.2) | 18.5 (15.9, 21.1) | -2.2 (-5.1, 0.7) | 0.134 |
| South Africa | 139 | 79 |  | ΔWT | 5.3 (5.0, 5.5) | -- |  |  |
|  |  |  |  | ΔFM | 1.5 (1.3, 1.7) | 29.1 (26.2, 32.0) | 8.4 (5.1, 11.6) | <0.001 |
| Sri Lanka | 68 | 40 |  | ΔWT | 5.1 (4.8, 5.5) | -- |  |  |
|  |  |  |  | ΔFM | 0.6 (0.3, 0.8) | 10.9 (6.7, 15.1) | -9.9 (-14.1, -5.6) | <0.001 |

Abbreviations: fat mass (FM), weight (WT)

^a^Estimates are from seemingly unrelated regression models (outcomes = ΔWT and ΔFM) adjusted for sex (boys [referent], girls), country (Brazil [referent], Pakistan, South Africa, Sri Lanka), decimal months between the first and last measurements (centered at the mean), and WT or FM at the first measurement (centered at the mean).

Supplementary Table 7. Descriptive statistics for the four groups of infants shown in Supplementary Figure 1, Panel A

|  |  | Deuterium Dilution sample (3-24 months) | | | | |
| --- | --- | --- | --- | --- | --- | --- |
|  |  | Total  (N=555) | BMI increasing,  FMI increasing  (N=94, 16.9%) | BMI increasing,  FMI decreasing  (N=38, 6.9%) | BMI decreasing,  FMI increasing  (N=135, 24.3%) | BMI decreasing,  FMI decreasing  (N=288, 51.9%) |
| Sex |  |  |  |  |  |  |
| Boys | N (%) | 277 (49.9) | 39 (41.5) | 18 (47.4) | 70 (51.9) | 150 (52.1) |
| Girls | N (%) | 278 (50.1) | 55 (58.5) | 20 (52.6) | 65 (48.2) | 138 (47.9) |
| Country |  |  |  |  |  |  |
| Brazil | N (%) | 216 (38.9) | 35 (37.2) | 15 (39.5) | 65 (48.2) | 101 (35.1) |
| Pakistan | N (%) | 132 (23.8) | 25 (26.6) | 14 (36.8) | 25 (18.5) | 68 (23.6) |
| South Africa | N (%) | 139 (25.1) | 24 (25.5) | 5 (13.2) | 32 (23.7) | 78 (27.1) |
| Sri Lanka | N (%) | 68 (12.3) | 10 (10.6) | 4 (10.5) | 13 (9.6) | 41 (14.2) |
| WT gain |  |  |  |  |  |  |
| Slow (< -0.67 Z-scores) | N (%) | 111 (20.0) | 1 (1.1) | 1 (2.6) | 19 (14.1) | 90 (31.3) |
| Normal | N (%) | 313 (56.4) | 28 (29.8) | 14 (36.8) | 91 (67.4) | 180 (62.5) |
| Rapid (> +0.67 Z-scores) | N (%) | 131 (23.6) | 65 (69.2) | 23 (60.5) | 25 (18.5) | 18 (6.3) |
| Length growth |  |  |  |  |  |  |
| Slow (< -0.67 Z-scores) | N (%) | 108 (19.5) | 20 (21.3) | 10 (26.3) | 19 (14.1) | 59 (20.5) |
| Normal | N (%) | 266 (47.9) | 36 (38.3) | 20 (52.6) | 70 (51.9) | 140 (48.6) |
| Rapid (> +0.67 Z-scores) | N (%) | 181 (32.6) | 38 (40.4) | 8 (21.1) | 46 (34.1) | 89 (30.9) |
| First measurement |  |  |  |  |  |  |
| 3 months | N (%) | 451 (81.3) | 88 (93.6) | 30 (79.0) | 117 (86.7) | 216 (75.0) |
| 6 months | N (%) | 83 (15.0) | 5 (5.3) | 7 (18.4) | 16 (11.9) | 55 (19.1) |
| 9 months | N (%) | 21 (3.8) | 1 (1.1) | 1 (2.6) | 2 (1.5) | 17 (5.9) |
| Last measurement |  |  |  |  |  |  |
| 15 months | N (%) | 16 (2.9) | 6 (6.4) | 0 | 5 (3.7) | 5 (1.7) |
| 18 months | N (%) | 83 (15.0) | 12 (12.8) | 11 (29.0) | 11 (8.2) | 49 (17.0) |
| 24 months | N (%) | 456 (82.2) | 76 (80.9) | 27 (71.1) | 119 (88.2) | 234 (81.3) |
| First measurement |  |  |  |  |  |  |
| WT Z-score | Mean (SD) | -0.29 (1.01) | -0.73 (0.97) | -0.48 (0.79) | -0.38 (0.95) | -0.09 (1.02) |
| Length Z-score | Mean (SD) | -0.55 (1.02) | -0.35 (1.01) | -0.16 (1.03) | -0.75 (1.01) | -0.56 (1.00) |
| WT (kg) | Mean (SD) | 6.28 (1.11) | 5.70 (0.81) | 6.13 (0.81) | 6.10 (0.97) | 6.57 (1.19) |
| FM (kg) | Mean (SD) | 1.42 (0.65) | 1.04 (0.47) | 1.52 (0.58) | 1.09 (0.44) | 1.70 (0.66) |
| BMI (kg/m^2^) | Mean (SD) | 16.85 (1.75) | 15.65 (1.45) | 16.04 (1.26) | 16.87 (1.50) | 17.33 (1.78) |
| FMI (kg/m^2^) | Mean (SD) | 3.78 (1.52) | 2.84 (1.21) | 3.94 (1.27) | 2.98 (1.05) | 4.44 (1.49) |
| Last measurement |  |  |  |  |  |  |
| WT Z-score | Mean (SD) | -0.19 (1.14) | 0.43 (1.13) | 0.24 (0.80) | -0.28 (1.07) | -0.40 (1.12) |
| Length Z-score | Mean (SD) | -0.32 (1.15) | -0.05 (1.12) | -0.34 (1.11) | -0.37 (1.25) | -0.39 (1.11) |
| WT (kg) | Mean (SD) | 11.48 (1.62) | 12.24 (1.76) | 11.88 (1.27) | 11.39 (1.51) | 11.21 (1.58) |
| FM (kg) | Mean (SD) | 2.50 (0.92) | 3.01 (1.02) | 2.18 (0.63) | 2.71 (0.79) | 2.27 (0.88) |
| BMI (kg/m^2^) | Mean (SD) | 15.88 (1.48) | 16.75 (1.57) | 16.71 (1.36) | 15.72 (1.21) | 15.56 (1.44) |
| FMI (kg/m^2^) | Mean (SD) | 3.46 (1.26) | 4.14 (1.41) | 3.07 (0.90) | 3.76 (1.07) | 3.15 (1.21) |

Abbreviations: body mass index (BMI), fat mass (FM), fat mass index (FMI), weight (WT)

Supplementary Table 8. The proportion of BMI change due to FMI change between first and last measurements (3-24 months) in the Deuterium Dilution sample^a^

|  |  |  | Mean (kg/m^2^)  Estimate (95% CI) | Percentage  Estimate (95% CI) | Difference  Estimate (95% CI) P-value | |
| --- | --- | --- | --- | --- | --- | --- |
| Models 1 | Boys | ΔBMI | -0.7 (-0.9, -0.5) | -- |  |  |
|  |  | ΔFMI | -0.4 (-0.6, -0.3) | 63.7 (43.2, 84.1) | Referent |  |
|  | Girls | ΔBMI | -0.7 (-0.9, -0.6) | -- |  |  |
|  |  | ΔFMI | -0.2 (-0.3, 0.0) | 25.7 (7.7, 43.6) | -38.0 (-59.5, -16.5) | 0.001 |
| Models 1 | Brazil | ΔBMI | -0.7 (-0.9, -0.5) | -- |  |  |
|  |  | ΔFMI | -0.4 (-0.6, -0.3) | 63.7 (43.2, 84.1) | Referent |  |
|  | Pakistan | ΔBMI | -1.2 (-1.5, -1.0) | -- |  |  |
|  |  | ΔFMI | -0.9 (-1.0, -0.7) | 69.1 (55.2, 83.0) | 5.5 (-16.2, 27.2) | 0.622 |
|  | South Africa | ΔBMI | -0.8 (-1.0, -0.5) | -- |  |  |
|  |  | ΔFMI | 0.4 (0.2, 0.6) | -56.3 (-95.1, -17.4) | -119.9 (-163.8, -76.0) | <0.001 |
|  | Sri Lanka | ΔBMI | -1.5 (-1.9, -1.2) | -- |  |  |
|  |  | ΔFMI | -1.5 (-1.7, -1.2) | 95.1 (78.3, 111.9) | 31.4 (6.9, 56.0) | 0.012 |
| Models 2 | Slow WT gain (< -0.67 Z-scores) | ΔBMI | -2.0 (-2.2, -1.7) | -- |  |  |
|  |  | ΔFMI | -0.9 (-1.2, -0.7) | 46.6 (35.6, 57.6) | -6.7 (-19.8, 6.5) | 0.320 |
|  | Normal | ΔBMI | -1.0 (-1.2, -0.8) | -- |  |  |
|  |  | ΔFMI | -0.5 (-0.7, -0.4) | 53.3 (38.7, 67.9) | Referent |  |
|  | Rapid WT gain (> +0.67 Z-scores) | ΔBMI | 0.2 (-0.1, 0.4) | -- |  |  |
|  |  | ΔFMI | 0.0 (-0.2, 0.2) | -17.8 (-158.6, 123) | -71.1 (-217.4, 75.3) | 0.341 |
| Models 3 | Slow length growth (< -0.67 Z-scores) | ΔBMI | -0.4 (-0.8, -0.1) | -- |  |  |
|  |  | ΔFMI | -0.5 (-0.8, -0.3) | 125.9 (48.9, 202.9) | 61.1 (-9.0, 131.2) | 0.088 |
|  | Normal | ΔBMI | -0.7 (-0.9, -0.5) | -- |  |  |
|  |  | ΔFMI | -0.5 (-0.6, -0.3) | 64.8 (41.9, 87.6) | Referent |  |
|  | Rapid length growth (> +0.67 Z-scores) | ΔBMI | -0.7 (-0.9, -0.5) | -- |  |  |
|  |  | ΔFMI | -0.4 (-0.6, -0.2) | 56.6 (33.9, 79.4) | -8.2 (-31.4, 15.1) | 0.491 |

Abbreviations: body mass index (BMI), fat mass index (FMI), weight (WT)

^a^Estimates are from seemingly unrelated regression models (outcomes = ΔBMI and ΔFMI) adjusted for sex (boys [referent], girls), country (Brazil [referent], Pakistan, South Africa, Sri Lanka), decimal months between the first and last measurements (centered at the mean), and BMI or FMI at the first measurement (centered at the mean).

Supplementary Table 9. The proportion of BMI change due to FMI change in the Deuterium Dilution sample: analysis of country differences restricting the sample to infants whose first measurement was at 3 months and whose last measurement was at 24 months^a^

|  | Full sample  N | Restricted sample  N |  |  | Mean (kg/m^2^)  Estimate (95% CI) | Percentage  Estimate (95% CI) | Difference  Estimate (95% CI) P-value | |
| --- | --- | --- | --- | --- | --- | --- | --- | --- |
| Brazil | 216 | 150 |  | ΔBMI | -0.6 (-0.8, -0.4) | -- |  |  |
|  |  |  |  | ΔFMI | -0.2 (-0.4, 0.0) | 31.4 (6.1, 56.8) | Referent |  |
| Pakistan | 132 | 99 |  | ΔBMI | -1.0 (-1.3, -0.8) | -- |  |  |
|  |  |  |  | ΔFMI | -0.4 (-0.7, -0.2) | 42.8 (25.8, 59.9) | 11.4 (-16.2, 39.0) | 0.418 |
| South Africa | 139 | 79 |  | ΔBMI | -0.8 (-1.1, -0.5) | -- |  |  |
|  |  |  |  | ΔFMI | 0.5 (0.2, 0.7) | -59.3 (-104.3, -14.3) | -90.7 (-140.8, -40.6) | <0.001 |
| Sri Lanka | 68 | 40 |  | ΔBMI | -1.4 (-1.8, -1.0) | -- |  |  |
|  |  |  |  | ΔFMI | -1.1 (-1.4, -0.8) | 75.0 (55.1, 94.8) | 43.5 (13.7, 73.3) | 0.004 |

Abbreviations: body mass index (BMI), fat mass index (FMI), weight (WT)

^a^Estimates are from seemingly unrelated regression models (outcomes = ΔBMI and ΔFMI) adjusted for sex (boys [referent], girls), country (Brazil [referent], Pakistan, South Africa, Sri Lanka), decimal months between the first and last measurements (centered at the mean), and BMI or FMI at the first measurement (centered at the mean).

Supplementary Table 10. The estimated interaction between infant feeding and weight gain (on the proportion of weight change due to fat mass change) in the Air-Displacement Plethysmography sample (0-6 months)^a^

|  | Percentage of  ΔWT due to ΔFM | |
| --- | --- | --- |
|  | B (95% CI) | P-value |
| Infant feeding |  |  |
| Not exclusively breastfed at 3 months | Referent |  |
| Exclusively breastfed at 3 months | 2.28 (-0.25, 4.81) | 0.077 |
| WT gain |  |  |
| Slow (< -0.67 Z-scores) | -6.59 (-10.28, -2.91) | <0.001 |
| Normal | Referent |  |
| Rapid (> +0.67 Z-scores) | 5.03 (2.34, 7.71) | <0.001 |
| Interactions |  |  |
| Slow, exclusively breastfed | 4.77 (0.34, 9.20) | 0.035 |
| Rapid, exclusively breastfed | -0.14 (-3.68, 3.40) | 0.937 |

Abbreviations: fat mass (FM), weight (WT)

^a^Estimates are from regression models (outcome = percentage of ΔWT due to ΔFM) adjusted for sex (boys [referent], girls), country (Australia [referent], India, South Africa), decimal months between the first and last measurements (centered at the mean), and percentage body fat at the first measurement (centered at the mean).

**Supplementary Figure 1.** Participant flow chart


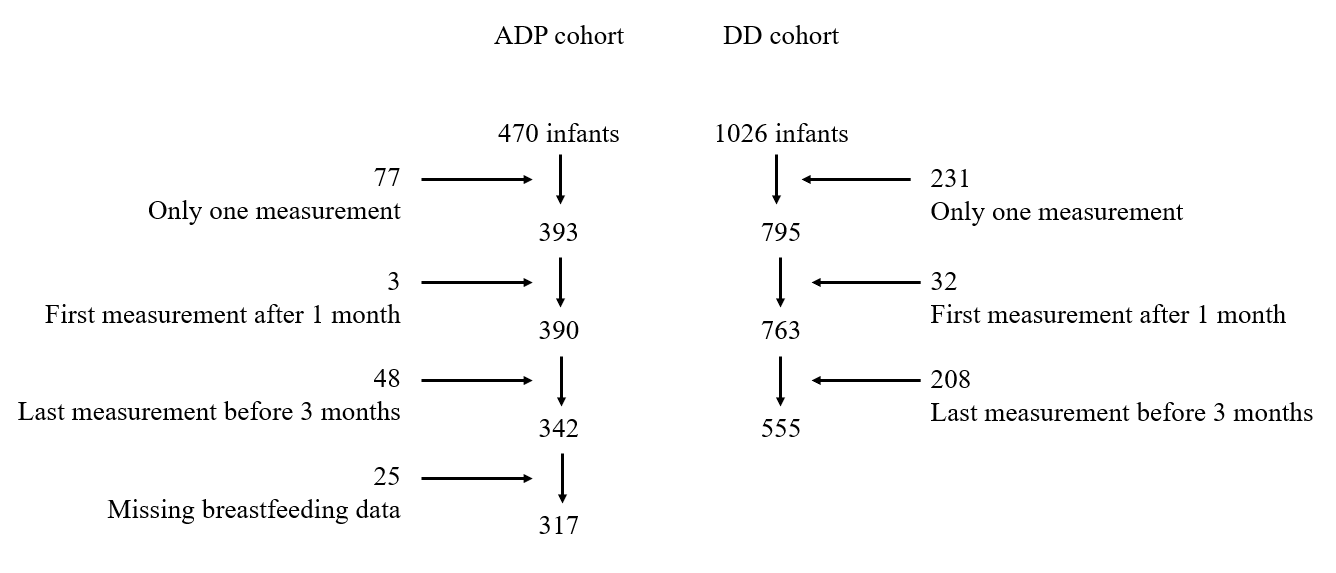


**Supplementary Figure 2.** Description of FMI change relative to BMI change in the Deuterium Dilution (3-24 months) sample.

Panel A. Paired co-ordinate arrow plot. Each line shows the data of one infant, connecting their first measurements to their last measurements (i.e., y1, x1 to y2, x2).

Panel B. Kernel density estimate plot. Excludes 18 infants with more extreme values (<-700% or >1300%).

Abbreviations: body mass index (BMI), fat mass index (FMI)

**
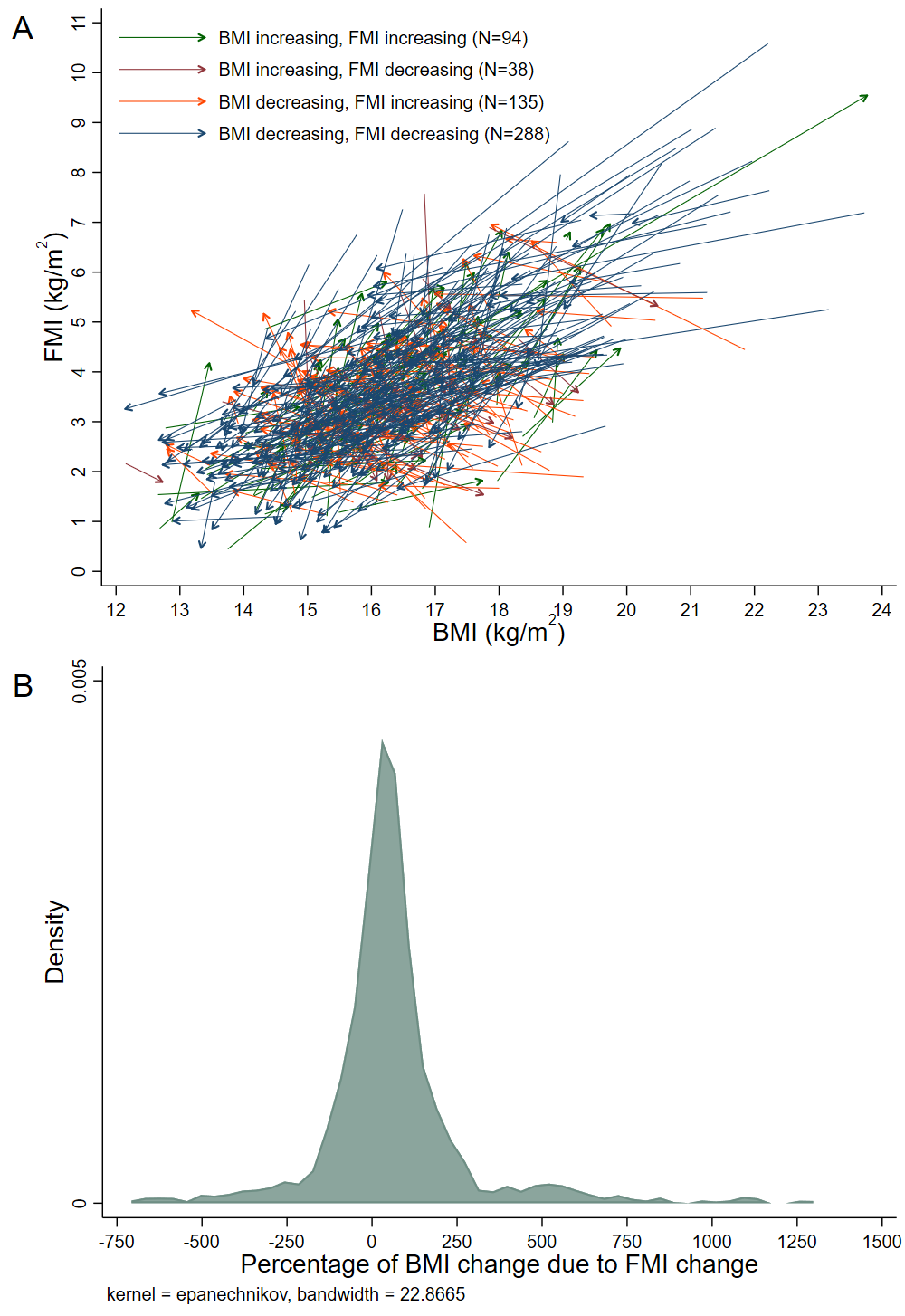
**

**Supplementary Figure 3.** The interaction between infant feeding and weight gain (on the proportion of weight change due to fat mass change) in the Air-Displacement Plethysmography sample (0-6 months).

Corresponds to the model in Supplementary Table 10.


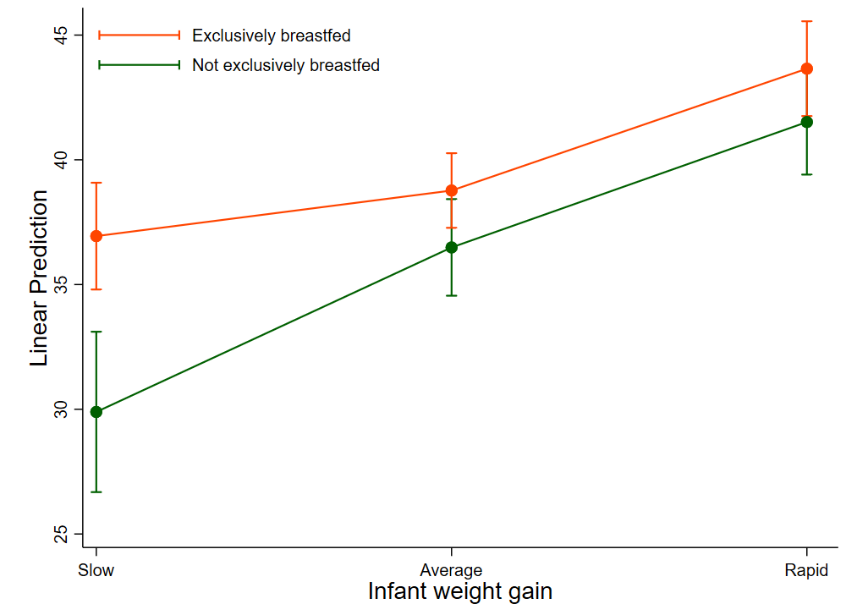

Supplement: Supplementary file 1 — Supplementary Material [file 41430_2024_1534_MOESM1_ESM.docx]
